# Infants and the decision to provide ongoing child welfare services

**DOI:** 10.1186/s13034-017-0162-7

**Published:** 2017-05-01

**Authors:** Joanne Filippelli, Barbara Fallon, Nico Trocmé, Esme Fuller-Thomson, Tara Black

**Affiliations:** 1 Factor-Inwentash Faculty of Social Work, University of Toronto, 246 Bloor Street West, Toronto, ON M6S 3W6 Canada; 2 McGill University, 3506 University Street, Room#301, Montreal, QC H3A 2A7 Canada

**Keywords:** Infants, Child welfare services, Decision-making, Child maltreatment

## Abstract

**Background:**

Infants are the most likely recipients of child welfare services; however, little is known about infants and families who come into contact with the child welfare system and factors that are associated with service provision. Investigations involving infants and their families present an unparalleled opportunity for the child welfare sector to enhance infants’ safety and well-being through early identification, referral and intervention. Understanding how the child welfare system responds to the unique needs of infants and caregivers is critical to developing appropriate practice and policy responses within the child welfare sector and across other allied sectors. This study examines maltreatment-related investigations in Ontario involving children under the age of one to identify which factors are most influential to predicting service provision at the conclusion of a child welfare investigation.

**Methods:**

A secondary analysis of the fifth cycle of the Ontario Incidence Study of Reported Child Abuse and Neglect (OIS) for 2013 was conducted. The OIS is a cross-sectional child welfare study that is conducted every 5 years. The most influential factors that were associated with the decision to transfer a case to ongoing services were explored through a multivariate tree-classification technique, Chi square automatic interaction detection.

**Results:**

There were an estimated 7915 maltreatment-related investigations involving infants in 2013. At least one caregiver risk factor was identified in approximately three-quarters (74%) of investigations involving infants. In the majority of investigations (57%), at least one referral for specialized services was provided. Primary caregiver with few social supports was the most highly significant predictor of the decision to provide ongoing child welfare services. Primary caregiver risk factors were predominant in this model. The analysis identified subgroups of investigations involving infants for which the likelihood of being transferred to ongoing services ranged from approximately 11–97%.

**Conclusion:**

Caregivers of infants are struggling with numerous challenges that can adversely compromise their ability to meet the unique developmental needs of their infant. The findings underscore the importance of community and social supports in decision-making.

## Background

Infants under the age of one constitute one of the most vulnerable groups of children in Canada. Compared to all other age groups of children, infants are the most likely to: be investigated [1]; be substantiated for maltreatment [2]; be the recipients of ongoing child welfare services [1]; be placed out-of-home [1], and be re-reported [3]. As a result of their physical vulnerability, infants are also the most likely to suffer injury and death as a consequence of maltreatment [4, 5]. The research amassed to date from a variety of disciplines, underscores that the rapid rate of brain development leaves infants particularly susceptible to the deleterious consequences of maltreatment on brain architecture and on various developmental domains, including cognitive and socio-emotional (e.g., [6–8]). Moreover, a growing and compelling body of research is highlighting the association between early adverse childhood experiences, such as maltreatment, and the increased risk of poor mental and physical health outcomes across the life course [9], including heart disease [10] cancer [11] depression [12], and premature mortality [13].

Child welfare decisions made on behalf of infants are complex, high stakes, and have consequences that can reverberate throughout an infant’s lifetime. Maltreatment that begins in infancy is likely to become chronic if infant and family needs remain unaddressed and appropriate interventions are not implemented [3]. Investigations of reported child abuse and neglect present the child welfare system and its community partners with an unmatched opportunity to target interventions [14]. Timely identification and intervention are critical because the developing brain is most responsive to environmental input and experiences in early childhood [15, 16]. Yet, there have been long-standing concerns with the adequacy of the child welfare system’s response to the distinct needs of infants, prompting calls for a more developmentally driven approach to child welfare practice, policy and research [8, 17, 18].

Although the notions of protection and well-being are central considerations in provincial and territorial statutes across Canada [19], child welfare services have traditionally placed greater emphasis on protection, including case identification and the investigation of maltreatment, in comparison to assessing child functioning and the quality of the infant-caregiver relationship [9, 20]. There are three key challenges that emanate from the literature with respect to meeting the unique needs of infants within the context of traditional child protection frameworks and service delivery models: (1) the large proportion of investigations involving infants where there is no specific incident of maltreatment alleged; (2) the disproportionate developmental vulnerability of infants to the impact of maltreatment and other adversities; and, (3) the intrinsic link and impact of caregiver functioning and the quality of the infant-caregiver relationship to infants’ immediate and long-term development and well-being [21].

Changes to the child welfare legislation and practices in Ontario have broadened the investigation mandate, contributing to the increased identification of vulnerable infants to the child welfare system for reasons other than specific incidents of alleged maltreatment [19, 22, 23]. The 2008 cycle of the Ontario Incidence Study of Reported Child Abuse and Neglect (OIS-2008) was the first to track risk-only investigations in the province [2]. In the OIS-2008 analysis of infant investigations by Fallon and colleagues [22], just over two-thirds (66.6%) of all infant investigations were categorized as either risk-only or as cases where children had been exposed to intimate partner violence (IPV). The exposure of children to IPV has been noted as the fastest growing type of investigated maltreatment in Canada [20]. Canadian research has identified opportunities for implementing alternative or differential responses to child protection in situations where IPV is the sole concern, as in these circumstances, investigations are less likely to lead to ongoing services, court involvement, or child welfare placement [20, 24, 25].

Infants and young children involved with the child welfare system have also been found to present with higher rates of developmental delays than children in the general population [26, 27]; yet, in comparison to other age groups, functioning concerns involving infants (e.g., attachment issues, intellectual/developmental delays, failure to meet developmental milestones, Fetal Alcohol Syndrome/Fetal Alcohol Effect, positive toxicology at birth, and physical disability) have consistently been less frequently identified in provincial and Canadian incidence studies [22]. The under-identification of infant functioning concerns may be a consequence of several possible reasons, including the lack of routine detailed child functioning assessments during initial investigations and the absence of appropriate measures and validated measurement instruments with established population norms for child functioning concerns [2, 22, 23]. There are also other challenges associated with reliably identifying child functioning concerns in infants without the assistance of standardized measures or direct assessment; for instance, caregivers may be hesitant in reporting their child’s functioning issues as a result of perceived consequences, such as blame [28]. There is minimal research that provides guidance with respect to the implementation of developmental screening and assessment practices and models for infants and young children within a child welfare context; for example, eligibility criteria for screening, which standardized measures to use, the timing, the frequency of use, and which professional should conduct the assessment [29, 30]. Yet, despite these challenges, the monitoring of developmental outcomes by the child welfare system has been identified as critical to addressing and evaluating child well-being, a central component of the child welfare mandate [29].

Despite the vast majority of children remaining in the home following a child protection investigation [2, 31], most research on child welfare decision-making has been focused on the decision to place children out-of-home [e.g., 23, 32, 33]. The body of literature focusing on the decision to provide ongoing child welfare services after a child maltreatment investigation is minimal [e.g., 32, 33] and predominantly originates in the United States. The decision to substantiate has been found to be associated with the decision to provide ongoing services [34, 35]. Younger caregiver age, the presence of caregiver impairments, caregiver history of past maltreatment, greater number of children in the home, maternal alcohol and drug use have been associated with the child welfare service provision decisions [34]. Younger children (<5 years) have also been found to be more likely to receive child welfare services than older children [35]. Jonson-Reid examined the likelihood of a case being opened for services post-investigation among older children and found that early adolescents (ages 11–14 years) had a higher likelihood of a case opening following an investigation than other age groups (7–10 year olds and 15–17 year olds) [32]. Ethnicity and race have also been found to impact case opening and service provision decisions [32, 36]. For instance, African American children were more likely to have a case opened than non-Hispanic White children [32]. Maltreatment type has also influenced the decision to provide ongoing services, as children who were reported for sexual abuse and who were under 14 years of age were more likely to have a case opening than children who were reported for physical abuse or neglect [32]. Similarly, families with allegations of neglect, emotional abuse, and infants exposed to substances have been found to be most likely to receive in-home child welfare services [35]. In addition, families with allegations that their children were “at-risk of harm” have been noted to be more likely to receive services when compared to those without these allegations [35].

Although evolving, research on the decision to provide ongoing child welfare services within a Canadian context is limited. Fallon, Ma, Black and Wekerle examined the characteristics of young parents who were investigated and had their cases opened for ongoing child welfare services and found that primary caregiver risk factors (e.g., few social supports, drug/solvent abuse, cognitive impairment, mental health issues, and physical health issues) were the most important predictors associated with the decision to provide ongoing services [14]. Intimate partner violence (IPV) was not a significant factor associated with the decision to provide ongoing child welfare services. Similarly, caregiver risk factors (e.g., few social supports, younger caregiver age) were significantly associated with the likelihood of receiving ongoing services or a referral to specialized services in Jud, Fallon, and Trocmé’s study [33]. Moreover, male gender, substantiated risk investigations, and suspected or substantiated investigations of exposure to intimate partner violence (IPV) were most likely to receive a service when compared to other investigations. Fast, Trocmé, Fallon, and Ma explored the decision to provide ongoing services in a Canadian sample of adolescents and found that internalizing functioning concerns (e.g., depression/anxiety/withdrawal, suicidal thoughts, self-harming behavior) was the most significant predictor of this decision [1]. Investigations involving Aboriginal children, the identification of caregiver risk factors, and the identification of household risk factors increased the likelihood of the provision of ongoing child welfare services [1].

Only two studies have specifically focused on exploring the factors associated with the decision to provide ongoing child welfare services to infants (under the age of one) and their families post-investigation; both are Canadian and utilized national and provincial datasets, respectively [22, 37]. In both studies, caregiver risk factors drove the decision to provide ongoing services, with differing risk factors emerging as a result of the source of the referral (i.e., hospital, police, non-professional sources, and/or community services or social service sources). Caregiver risk factors, including the presence of a cognitive impairment, victim of intimate partner violence, few social supports, drug/solvent abuse, mental health issues, and younger caregiver age (under 21), significantly increasing the likelihood of ongoing service provision in both studies.

The state of the current literature highlights both the complexity and challenges in understanding the decision to provide ongoing child welfare services. The body of literature that focuses on the decision to provide post-investigation child welfare services is highly varied. There are substantial variations with respect to: the definition of ongoing services, type and measurement of variables examined, the sample and sample size, methodology, the time period for data collection, and the country of study origin. The lack of consistency across studies makes it difficult to bring cohesion to this body of literature with respect to the ongoing service provision decision and its’ associated factors. The majority of the literature originates from the United States and does not focus on infants specifically. Several factors act to limit the comparability and generalizability of findings from the broader literature to the Ontario context, including differences in service models (e.g. implementation of differential response, funding, policy, and legislative mandates). For instance, reports that allege risk of harm are not accepted in all states in the US [35]. The broader environmental context between Canada and the US differs. In comparison to the US, Canada has lower child poverty rates, lower rates of teen parenting, universal health care, and a broader network of social support programs [38–40].

Research exploring infants and the child welfare system’s service provision decisions is underdeveloped, underscoring the need to advance the current knowledge base. When compared to all other age groups in Canada, infants have been found to be the most likely to receive post-investigation services [1]. The decision to provide ongoing child welfare services at the conclusion of an investigation has enormous implications for infants, their families, and for the child welfare system itself. For instance, the decision to provide ongoing services has financial implications for child welfare organizations that operate in an environment of ongoing fiscal restraint [14].

The present analysis uses the Ontario Incidence Study of Reported Child Abuse and Neglect-2013 (OIS-2013) [23] to examine the profile of infants and families reported and investigated by the child welfare system across Ontario in a representative sample of child welfare organizations. Factors associated with ongoing child welfare service provision will also be examined. The research questions are:

1. What are clinical characteristics (child, primary caregiver, household, case, and service) of maltreatment-related investigations involving infants in Ontario in 2013?
2. What characteristics influence and are most predictive of the decision to transfer a case to ongoing child welfare services in Ontario in 2013?

This study seeks to contribute and extend the knowledge base concerning infants and the decision to provide ongoing child welfare services after a referral for a child maltreatment-related concern. There is a dearth of research on how the child welfare sector in Canada responds to infants. This study utilizes a multivariate tree-classification technique for analyzing decision-making influences. Tree classification approaches have been rarely used in the field of child welfare, but are promising in terms of developing a more nuanced understanding of decision-making influences that could be overlooked by more traditional multivariate techniques by ranking the influences of each independent variable and assessing how multiple independent variables interact or work together to predict the service provision decision [41]. Lastly, in contrast to the only previous study in Ontario regarding infants [22], this study includes child ethnicity and child sex variables in the model, which can be key drivers in the decision to provide ongoing services. A greater understanding of the profile of infants and their families and the factors that influence the child welfare system’s decision to provide post-investigation services is critical to strategically developing appropriate practice and policies that are aligned with their unique needs.

## Methods

A secondary analysis of the fifth cycle of the Ontario Incidence Study of Reported Child Abuse and Neglect (OIS-2013) was conducted. The primary objective of the OIS-2013 was to examine provincial estimates on the incidence of reported child maltreatment, and the characteristics of the children and families investigated by the child protection system in Ontario.

### Sample

The OIS utilized a three-stage sampling design to select a representative sample of 17 child welfare agencies from a provincial list of 46 in Ontario [23]. A random sample of Ontario child welfare agencies was selected. A sample of cases was then selected from within each of these sampled agencies. Cases opened between October 1, 2013 and December 31, 2013 of the study cycle were eligible for inclusion in the study. Children not reported to child welfare services, screened out reports, and new allegations on open cases at the time of selection were not included in the OIS-2013. The 3-month study period is considered optimal for participation and compliance with study procedures. It is noteworthy that in Ontario cases are counted as families. There were 3118 cases opened at the family-level during the 3-month period. The final stage of the sampling consisted of identifying investigated children as a result of maltreatment concerns. There were a total of 5265 children investigated as a result of the identification of maltreatment concerns. Of those 5265 children investigated, 345 were infants. Maltreatment-related investigations included in the OIS-2013 are comprised of two types of investigations: (1) where there is no specific concern about past maltreatment but future risk of maltreatment is being assessed (risk-only), and, (2) investigations where maltreatment may have occurred. Both types of maltreatment-related investigations, regardless of their substantiation status were included in this analysis. Children over 15 years of age, siblings who were not investigated, and children who were investigated for non-maltreatment concerns were excluded from the sample.

Provincial estimates were derived by applying full weights, which includes both annualization and regionalization weights. These procedures yielded a final weighted sample of 125,281 children investigated because of maltreatment concerns. This study focused specifically on maltreatment-related investigations involving infants (under the age of 1 year) and explored predictors associated with the decision to provide ongoing child welfare services at the conclusion of the investigation. The final provincial estimate was 7915 investigations involving infants.

### Data collection instrument

Data for the OIS-2013 is collected using a three-page standardized data collection instrument: the Maltreatment Assessment Form. The primary investigating child welfare worker completed this form at the conclusion of the child protection investigation. The instrument collected clinical information that child welfare workers routinely collect as part of their initial investigation, such as: infant, caregiver, case characteristics, and short-term service dispositions. A reliability study of the Maltreatment Assessment Form was conducted and the majority of items demonstrated good to excellent test re-test reliability [23].

### Measures

#### Outcome variable: transferred to ongoing service

Workers were asked to indicate whether the case would be opened for ongoing child welfare services at the conclusion of the intake investigation. The decision to transfer a case to ongoing services is dichotomous. The variable definitions and codes used in this analysis are provided in Table 1.

**Table 1:** Variable definitions and codes

Variable	Description	Measurement
Outcome	Workers were asked if they planned to keep the case open to provide ongoing services to the family at the conclusion of the investigation	Dichotomous variable: 1 Transfer to ongoing child welfare services 0 Case closure
Predictors		
Child characteristics		
Child sex	Worker identified the sex of the investigated child	Dichotomous variable: 1 Male 0 Female
Child functioning	Workers were asked to note up to eighteen child functioning concerns. Six of eighteen dichotomous child functioning variables were relevant to infants: failure to meet developmental milestones, attachment issues, intellectual/developmental disability, FAS/FAE, positive toxicology at birth, and physical disability. This analysis noted whether the worker examined at least one of six of these relevant concerns	1 At least one child functioning concern noted (suspected or confirmed) 0 No child functioning concerns noted
Child ethnicity	Workers were asked to indicate the ethnicity of the child (Black, Latin American, Arab, Aboriginal, Asian). Ethno-racial categories developed by Statistics Canada	Dichotomous variable: 1 Ethnic minority 0 White
Caregiver Characteristics		
Primary caregiver age	Workers were asked to indicate the age category of the primary caregiver.	Categorical variable: 1 18 years and under 2 19–21 years 3 22–30 years 4 31–40 years 5 41 years and up
Primary caregiver risk factors	Workers could note up to nine risk factors for the primary caregiver. Concerns were: alcohol abuse, drug/solvent abuse, cognitive impairment, mental health issues, physical health issues, few social supports, victim of domestic violence, perpetrator of domestic violence, and history of foster care/group home	Nine dichotomous variables: 1 Suspected or confirmed caregiver risk factors 0 No caregiver risk factors noted
Primary income of caregiver	Workers were asked to indicate the primary source of the primary caregiver’s income	Categorical variable: 1 Full time 2 Part-time/seasonal 3 Other benefits/unemployment 4 No income
No second caregiver in the home	Workers were asked to describe up to two caregivers in the home. If there was only one caregiver described, it was assumed there was no second caregiver in the home	Dichotomous variable: 1 No second caregiver in home 0 Second caregiver in home
Household hazards	Workers were asked to note if the following hazards were present in the home at the time of the investigation: accessible weapons, accessible drugs, production/trafficking of drugs, chemicals/solvents, used in drug production, other home injury hazards, and other home health hazards	Dichotomous variable: 1 At least one household hazard 0 No household hazard
Household regularly runs out of money for basic necessities	Workers were asked to note if the household regularly runs out of money for basic necessities including food, shelter and clothing	Dichotomous variable: 1 Family regularly ran out of money 0 Family did not regularly run out of money
Number of moves	Workers were asked to note the number of moves the household had in the past 6 months	2 2 or more moves 1 One move 0 None
Case characteristics		
Previous openings	Worker indicated if there were one or more previous child protection openings	1 One or more previous openings 0 No previous openings
Type of investigation	Workers were asked to indicate if the investigation was conducted for a specific maltreatment incident, or if it was to assess a risk of maltreatment only	1 Maltreatment investigation 2 Risk-only investigation

### Predictor variables

Informed by a bioecological model of human development, clinical variables were chosen based on their availability and the empirical literature on factors related to child maltreatment, its’ consequences, and child welfare system’s response to infants and their families.

### Statistical analysis plan

All analyses were conducted using SPSS version 23. Descriptive analyses were conducted to explore the profile of all infant maltreatment-related investigations. The multivariate tree classification technique, Chi square automatic interaction detection (CHAID) was utilized for data analysis in order to identify factors predictive of the decision to transfer a case to ongoing child welfare services at the conclusion of the intake investigation. CHAID was deemed an appropriate statistical method as this study is exploratory in nature and CHAID is considered an exploratory data analysis technique [41].

CHAID has been used to explore differences in groups based on categorical predictor variables. Predictors are split into categories based on the Chi square statistic. CHAID selects the predictors that divide the sample into subsets or segments that are most different on the dependent variable. Additional predictors that most influence the classification of the dependent variable then successively split each subset created by the initial split. The segmentation process is based on interactions between predictors [41]. Each group created by a split is independently evaluated to determine the next best predictor. Splits are based on the combination of categories that maximize homogeneity within nodes (subgroups) and continue until no significant splits can be made or the node size is too small for further analyses. Each node is representative of a particular subgroup or subsample of maltreatment-related investigations involving infants.

Although tree-based analysis is uncommon in child welfare research, it has been recently utilized to explore child welfare service provision decisions with infants specifically in a Canadian child welfare context [22, 37]. There are numerous advantages noted in the literature in using tree classification techniques over other traditional statistical techniques. In comparison to descriptive statistics, Chi square analyses, and logistic regression, classification tree analysis can examine complex interactions between variables [41, 42].

This present analysis included infant (e.g. sex), caregiver (e.g., caregiver risk), household (e.g., household hazards), and case characteristics (e.g., previous openings). Please see Table 2 for all variables included in the model and the definitions of those variables. The model was developed in order to determine how various infant, caregiver, household and case characteristics interact and work together to predict the decision to provide ongoing services to infants and families following an initial investigation. In order to avoid over fitting the data, the minimum sizes for parent (n = 50) and child nodes (n = 20) was specified. Cross validation was utilized in order to assess the generalizability and stability of the final model. CHAID analysis classifies missing values for a particular variable as a unique category, which it subsequently collapses with other statistically homogenous categories [43]. Frequency counts were produced with study weights. The multivariate CHAID analysis was unweighted in order to examine the individual decisions at the worker level; thus, a sample of 345 infants was used for the CHAID analyses.

**Table 2:** Clinical profile of maltreatment-related investigations involving infants in Ontario in 2013 *Source*: 2013 Ontario Incidence Study of Reported Child Abuse and Neglect*

Variable	Estimate	%	% Transferred to ongoing services
Child characteristics			
Child sex			
Female	3887	49.3	45.9
Male	3999	50.7	34.1
Child ethnicity			
Ethnic minority	2396	30.9	29.5
White	5355	69.1	43.9
Child functioning concerns			
Attachment issues	–	0.8	100
Intellectual/developmental disability	–	0.6	73.9
Failure to meet developmental milestones	162	2.1	84.7
FAS/FAE	–	0.8	100
Positive toxicology at birth	305	3.9	87.5
Physical disability	124	1.6	50.0
At least one child functioning concern	614	7.8	78.5
Primary caregiver characteristics			
Primary caregiver age			
18 years and under	851	10.8	45.1
19–21 years	1616	20.5	59.7
22–30 years	3347	42.5	35.9
31–40 years	1952	24.8	28.1
41 years and up	112	1.4	14.3
Primary caregiver risk factors			
Alcohol abuse	842	10.6	77.1
Drug/solvent use	1490	18.8	76.6
Cognitive impairment	699	8.8	84.1
Mental health issues	2459	31.1	68.2
Physical health issues	466	5.9	68.2
Few social supports	2597	32.8	65.2
Victim of intimate partner violence (IPV)	2978	37.6	51.5
Perpetrator of domestic violence	854	10.8	51.3
History of foster care	1031	13.0	58.8
At least one caregiver risk factor	5883	74.3	50.3
Household characteristics			
No second caregiver in home	2403	30.4	54.1
Primary income			
Full-time	571	7.7	23.3
Part-time/seasonal	375	5.1	23.2
Other benefits/unemployment	5471	73.9	42.7
No income	1498	13.3	49.8
At least one household hazard	664	8.8	72.7
Household regularly runs out of money	1030	15.7	67.2
Number of moves			
No moves	3261	50.0	26.4
One move	2121	32.5	42.0
Two or more moves	1138	17.4	80.9
Maltreatment characteristics			
Types of maltreatment-related investigation			
Physical abuse	163	2.1	19.0
Sexual abuse	–	1.2	61.3
Neglect	1618	20.4	43.1
Emotional maltreatment	424	5.4	41.5
Exposure to IPV	2467	31.2	24.2
Risk	3150	39.8	50.6
Case characteristics and short-term service outcomes			
Opened for ongoing services	3151	39.8	–
At least one previous case opening (family-level)	3445	43.6	51.1
Reopened within 12-month period	2243	65.1	51.9
Infant previously investigated for alleged maltreatment	1339	16.9	44.6
At least one referral for specialized services	4530	57.2	57.1
Child welfare court	693	8.8	98.1
Placement	680	8.6	89.0

Estimated number of provincial investigations, n = 7915

– = not reportable because n < 100

## Results

### Profile of maltreatment-related investigations involving infants

Key descriptive information was revealed with respect to the characteristics of maltreatment-related investigations involving infants in Ontario in 2013, including information with respect to infant, family, maltreatment, household and case characteristics. The clinical profile of infant maltreatment-related investigations is presented in Table 2.

### Child and family characteristics

Half of maltreatment-related infant investigations involved infant males (50.7%, 3999 investigations). Approximately 30.9% (2056 investigations) of investigations were comprised of infants who were identified as ethnic minorities. At least one child functioning issue was noted in 7.8% of investigations (n = 614 investigations). The three most common concerns noted for infants by workers were positive toxicology at birth (3.9%, 305 investigations), followed by failure to meet developmental milestones (2.1%, 162 investigations) and physical disability (1.6%, 124 investigations).

Almost one-third of primary caregivers (31.3%, 2467 investigations) were under the age of 21 years. At least one primary caregiver risk factor was noted in 74.3% (5883 investigations) of investigations. The most common caregiver risk factor was victim of intimate partner violence (IPV) (37.6%, 2978 investigations), followed by caregiver with few social supports (32.8%; 2597 investigations) and caregiver mental health issues (31.1%, 2459 investigations).

### Household characteristics

Approximately a third of investigations involved single-parent households (30.4%, 2403 investigations). Almost three-quarters of caregivers (73.9%, 5471 investigations) involved in infant investigations were receiving benefits or unemployment (e.g. social assistance, employment insurance) as their primary source of income. In 13.3% (1498 investigations) of investigations, there was no reported income. A smaller minority of caregivers was identified as having either full-time (7.7%, 571 investigations) or part-time/seasonal employment (5.1%, 375 investigations). The presence of at least one household hazard (e.g., mold, inadequate heating) was noted in a small proportion of investigations (8.8%, 664 investigations). Half (50%, 3261 investigations) of investigated families had moved in the past year. One-third (32.5%, 2121 investigations) moved once and 17.4% (1138 investigations) of investigated families moved two or more times.

### Case characteristics and service dispositions

Out of a total of 7915 investigations involving infants, an estimated 39.8% (3151) of those investigations were transferred to ongoing child welfare services. Many investigations (43.7%, 3445 investigations) had at least one previous opening. Of those investigations with previous openings, almost two-thirds (65%, 2243 investigations) were reopened within 12 months of case closure. In the majority of infant investigations (57.2%, 4530 investigations), there was at least one referral for specialized services provided. The decision to initiate a court application was noted in the small minority of infant investigations (8.8%, 693 investigations), as was the decision to place out-of-home (8.6%, 680 investigations).

## Results of CHAID analysis

**Fig. 1:**
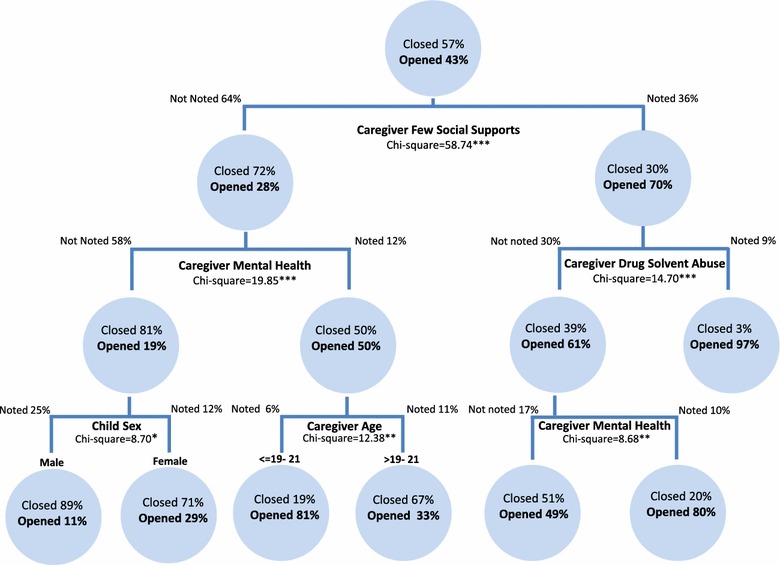
Transfers to ongoing child welfare service among all maltreatment-related investigations involving infants (n = 345)

To explore which predictors were most associated with the decision to provide ongoing child welfare services at the conclusion of the maltreatment-related investigation involving infants, a classification tree model was developed. The results of the model are illustrated in Fig. 1, including probability values that are adjusted for multiple comparisons using the Bonferroni approach. The resulting tree model predicted the decision to provide ongoing services among all infant investigations and consisted of 12 nodes (i.e., subgroup or subsample) across 3 levels. Predictors split each subgroup involving infants, subsequently resulting in the groupings of variables. A risk estimate of the cross-validation, a measure of the tree’s predictive accuracy was .307 (SE = .025), indicating that the category predicted by the model is incorrect for 30.7% of cases; whereas, the classification table indicates that the model classified 75.7% of infant investigations correctly.

Primary caregiver risk factors (e.g., few social supports, mental health issues, drug/solvent abuse, and primary caregiver age) dominated this model. Primary caregiver with few social supports emerged as the most highly significant predictor of the decision to provide ongoing child welfare services (χ^2^ = 58.74, df = 1, adj. p < .0001), as it was the first predictor selected by CHAID to split the sample of infant maltreatment-related investigations. Infant investigations where few social supports were noted by the investigating child welfare worker were more likely to be transferred to ongoing services in comparison to those that were not identified with this risk factor (70.2 vs. 27.6%).

CHAID revealed numerous interactions between independent variables that influenced the service provision decision. Among infant investigations where primary caregiver with few social supports was noted, the next best predictor of the investigation being transferred was primary caregiver drug/solvent abuse (χ^2^ = 14.70, df = 1, adj. p < .0001). Among this subsample, infant investigations where the primary caregiver was identified as having a drug/solvent abuse issue were more likely to be transferred than those who were not (96.9 vs. 60.9%). Among infant investigations where caregiver drug/solvent abuse was not noted, the next best predictor was primary caregiver mental health issues (χ^2^ = 8.68, df = 1, adj. p < .005).

Among infant investigations where primary caregiver with few social supports was *not noted*, the next best predictor of the transfer to ongoing services was primary caregiver mental health issues (χ^2^ = 20.67, df = 1 adj. p < .0001). Within this subsample, infant investigations with primary caregiver mental health issues identified were more than twice as likely to be transferred to ongoing services than investigations without this concern identified (50.0 vs. 19.3%). Among infant investigations where primary caregiver mental health issues was not noted, infant sex was the next best predictor (χ^2^ = 8.70, df = 1 adj. p < .05). More specifically, investigations involving female infants were more likely to be transferred than those involving males (28.9 vs. 10.6%). However, when mental health issues were noted, primary caregiver age was the next best predictor of ongoing issues (χ^2^ = 12.38, df = 1, adj. p < .005). The subsample containing infant investigations with primary caregivers under the age of 21 were more likely to be opened for ongoing services than those with primary caregivers 22 years of age or more (81.0 vs. 33.3%). The CHAID analysis collapsed the initial five different age categories into two separate subgroups. This suggests that the two initial age categories that formed the under 21 age group can be treated as homogenous, as can the three initial age categories that comprised the over 22 group. Infant sex was the only child characteristic that emerged as an influential predictor. Several child, household and case characteristics did not reach statistical significance in the model and were subsequently excluded.

The CHAID analysis identified subgroups of investigations involving infants for which the likelihood of being transferred to ongoing services ranged from 10.6 to 96.9%. The subgroup of infant investigations with the greatest likelihood of remaining open were those identified with primary caregiver risk factors of few social supports *and* drug/solvent abuse (96.9%). The subgroup of infant investigations where primary caregivers were below 21 years of age and identified mental health issues were also among the most likely subgroups to be provided ongoing services (81%). Overall, the CHAID analysis revealed the five following salient or significant factors in predicting the decision to transfer infant investigations for ongoing service provision: primary caregiver few social supports, primary caregiver mental health issues, child sex, primary caregiver age, and primary caregiver drug/solvent abuse.

## Discussion

This study used a Canadian provincial dataset to explore the clinical profile of infants and their families involved in maltreatment-related investigations and to determine which factors predict the provision of ongoing child welfare services at the conclusion of the investigation. Approximately 4 of every 10 investigations involving infants received ongoing services. Overall, the emerging clinical portrait of infant maltreatment-related investigations underscores the many burdens experienced by the infants and families that are investigated by the child welfare system. These findings provide a broad profile of the clinical factors that influence service provision and have practice, policy and research implications for the field of child welfare. This study both confirms and extends the knowledge base with respect to infants investigated by the child welfare system. In approximately 7 of every 10 infant investigations in Ontario, at least one primary caregiver risk factor was identified. Caregiver functioning issues have consistently emerged as driving service provision decisions for infants in a child welfare context [22, 37]. As the classification tree analysis underscored in this study, the most influential predictor of service provision was primary caregiver with few social supports. Other primary caregiver risk factors (i.e., mental health issues, younger caregiver age, and primary caregiver drug/solvent abuse) drove the decision to provide ongoing child welfare services in this analysis.

A classification tree approach allowed for a more nuanced understanding of the decision to provide ongoing services. This approach allowed for the exploration of interactions between predictors and the identification of mutually exclusive and exhaustive subgroups of investigations. Several independent variables worked together to predict the decision to provide ongoing services. For instance, subgroups of infant investigations where both primary caregiver with few social supports *and* the primary caregiver were identified as having a drug/solvent abuse issue were the most likely subgroup of investigations to remain open for ongoing services. The presence of a caregiver mental health issues had a prominent role in the model and worked in concert with other variables (e.g., few social supports) to increase the likelihood of service provision. It is notable that research on infant development indicates that in addition to maltreatment, other salient risk factors that can hinder optimal socio-emotional development are prenatal factors (e.g., exposure to drugs or alcohol), caregiver mental health factors (e.g., depression) and caregiver social factors (e.g., social isolation) [44]. Positive toxicology at birth was the most frequent (3.9%) child functioning concern noted by the investigating worker in this study. There is a large body of literature that suggests caregiver functioning issues, such as depression, may act to compromise the quality of the infant-caregiver relationship which is critical to ensuring the child’s safety and well-being [45]. This further highlights the important role of the child welfare system as a pathway to vulnerable connecting children and families to the right community supports and services [29].

It is notable that through the process of segmentation, the CHAID analysis revealed that within several seemingly high-risk subgroups some investigations were not being opened for ongoing services. Further research disentangling the possible differences between families that receive post-investigation child welfare services from those who do not is warranted. Understanding differences may assist in ensuring that scarce resources are appropriately targeted and aligned with infant and caregiver needs. Moreover, child welfare decisions have been conceptualized as being influenced by the interplay of complex factors at various levels including: case, worker, organizational, and environmental levels. An exploration of multi-level influences may help to better understand how these factors combine to influence the decision to provide ongoing child welfare services for infants.

The emergence of child sex as a significant predictor of service provision was an unexpected finding in this study. The incidence rates for maltreatment-related investigations involving infants are fairly similar for female and male infants in Ontario (58.88 vs 57.61 per 1000 children, respectively); yet, there was a larger proportion of female infants transferred to ongoing services when compared to male infants (45.9 vs 34.1%) at the conclusion of the maltreatment-related investigations. The Canadian research on child sex and service provision decisions is mixed. This study’s findings corroborate Jud, Fallon and Trocmé’s study indicating that child’s gender was significantly associated with the decision to provide ongoing services or a referral for specialized services for the family; more specifically, male children received fewer services than female children [33]. In contrast, Fast and colleagues did not find an association between child gender and the provision of ongoing service for adolescents in a Canadian sample [1]. Canadian studies that have examined the association between gender and placement [46] or the substantiation decision [47] in infant maltreatment investigations have not found a significant relationship at the bivariate level with an infant’s gender. Are the families of female infants perceived to be at greater risk and need of ongoing child welfare services than male infants? Understanding how gender influences the child welfare system’s response to infants and younger children is an important endeavor in light of the research that suggests that there are gender differences in the onset of early childhood mental health problems [48, 49]. There is also emerging evidence that the impact of early childhood maltreatment on behavioural problems seems to be modified by gender [50]. Further research is indicated with respect to the influence of child sex in maltreatment-related investigations and service provision decision-making involving infants.

Interestingly, although type of investigation (risk-only versus maltreatment) was not a significant predictor in this analysis, the proportion of investigations transferred to ongoing services that assessed risk exceeded investigations that assessed different types of maltreatment incidents (i.e., physical abuse, neglect, emotional maltreatment and IPV). This corroborates the literature that has found that investigations involving allegations of risk are more likely to be referred to services than other investigation types [33, 35]. Moreover, although infant functioning concerns were identified infrequently, they were transferred to ongoing services in high proportions. For instance, positive toxicology at birth was noted in 3.9% of investigations involving infants and these investigations were transferred approximately 87.5% of the time. Another noteworthy finding relates child ethnicity on the decision to provide services. Child ethnicity was not an influential predictor in this model. Recent Canadian studies have explored ethnicity and Aboriginal status specifically on the decision to transfer a case to ongoing services. Jud, Fallon and Trocmé found that ethnicity did not influence the decision to refer to specialized services [33]. Fast and colleagues found adolescents’ Aboriginal status did impact the decision to transfer to ongoing services [1]. This finding warrants future research specifically examining infants.

Among infant investigations with at least one previous case opening at the family level (43.7%), almost two-thirds were re-opened by the child welfare system within a 12-month period. Families that receive child welfare services have been found to be more likely to be re-reported than those who have not, raising concerns with respect to the effectiveness of child welfare interventions [35]. Moreover, 17.9% of infants involved in the index investigation had been previously investigated by the child welfare system for alleged maltreatment. High rates of re-reported maltreatment have been found in the literature among infants with both substantiated and unsubstantiated reports post-investigation [3]. Repeated child welfare involvement suggests that infants are at high risk of ongoing adversities [3] and is an important area for future research. The exposure of infants to chronic and excessive stress is believed to be “toxic” as it can adversely and permanently influence brain development [16, 51, 52]. Timely and appropriate interventions may decrease the likelihood that maltreatment beginning in infancy becomes chronic, adversely impacting development. These findings highlight the challenges of service provision and the importance of examining infants’ trajectories and child welfare service patterns (i.e. re-reports) over time in a Canadian context.

This analysis revealed that child welfare workers did not endorse the presence of any child functioning concerns in approximately 90% of all infant investigations in Ontario in 2013. Infant screening instruments can assist in identifying developmental risks in various developmental domains (e.g., motor, socio-emotional, cognitive) and in determining when referrals for further evaluation and intervention are needed [53]. There is no comprehensive or systematic developmental screening strategy in place in Ontario for infants involved with the child welfare system. Under-identification of infant functioning issues may be translating into missed opportunities for understanding, monitoring and enhancing developmental well-being and resilient functioning for infants involved with the child welfare system. Further research is required on developmental screening and assessment models for infants and young children who are involved with the child welfare system [30]. The lack of routine, detailed and systematic assessments of child functioning within a Canadian child welfare context would likely result in the identification of more issues for all children investigated [2]. Reliable and valid screening tools are important to this endeavor; however, in order for screening tools to be adopted and for models to be feasible and sustainable, the unique demands of child welfare settings must be considered. Moreover, the use of standardized tools would require training and/or close partnerships with professionals who are qualified to both administer and interpret the results of these instruments.

Risk, exposure to IPV and neglect were the three most common primary concerns noted in infant investigations. Risk investigations comprised the largest proportion of all infant investigations in this present analysis of the OIS-2013 and the previous OIS-2008 cycles, comprising 39.8 and 45.1%, respectively. The large proportion of risk-only investigations may be due to increasing professional awareness of the developmental impact of chronic exposure of family dysfunction and stress [19]. Exposure to IPV was identified as the primary concern in almost one-third of infant investigations and comprised the second largest proportion of investigations in this study. Canadian research is suggesting the importance of considering a differential systems response to IPV [19, 25]. Moreover, the child welfare response to IPV is dependent on the specific subtype of exposure to IPV being investigated (i.e., direct witness to physical violence, indirect exposure to physical violence, and exposure to emotional violence) [25]. Further research is needed to specifically explore the child welfare response to infants exposed to IPV and its subtypes. Lastly, neglect was the primary concern in 1 of every 5 infant investigations. Neglect has particularly pernicious effects on infants’ short-term and long-term well-being. Furthermore, neglect has been noted to be more chronic in duration and can result in a broader range of developmental harm in comparison to other maltreatment types, including language delays and deficits, cognitive deficits, impulsivity, and poor school performance [54].

In the majority (57.2%) of investigations involving infants, referrals were made for services. This finding further highlights the complexity of family needs and the importance of connecting families with additional supports and services in the community. The large proportion of referrals is not surprising given that caregiver with few social supports was the most influential predictor in the decision to provide ongoing services. The ability of the child welfare system to meet the needs of infants and families is tied to other systems. Caregiver isolation means less community surveillance for a very vulnerable population and may explain why infants are most likely to be transferred to ongoing services. Moreover, it is also possible that a transfer to ongoing services triggers other services for the family and may signal a lack of available and accessible community supports for caregivers and infants. Insight from the field, particularly from the perspective of child welfare workers with respect to decision-making and service provision is notably absent in the literature, but is viewed as necessary to advancing the knowledge base on decision-making processes, contexts and outcomes for infants.

Findings indicate that caregivers are struggling with numerous challenges that can adversely compromise their parenting and their ability to meet the unique developmental needs of their infant. Given that the protection and well-being of infants is intrinsically linked to their primary caregiver, exploration of how the child welfare system can simultaneously address and buffer infant and caregiver risk factors while promoting protective factors is critical. The infant-caregiver relationship is critical to promoting infant well-being.

Within the larger Canadian child welfare context, there has been a noted shift in the focus of investigations from immediate safety to the long-term impact of family dysfunction [19]. Yet, it appears that shifts in investigative focus have not been accompanied by shifts in child welfare service models. This raises questions about the utilization of alternative or differential response models or initiatives with respect to infants. Although the child welfare system in Ontario has shifted towards differential response options for the type of investigation conducted (i.e., forensic versus customized), Ontario has not yet implemented wide-scale programs [22, 55]. A comprehensive understanding of how the Canadian child welfare system is responding to infants is challenging to discern. Child welfare in Canada is governed by legislation that is specific to each province and territory [56, 57]. Although there are four other provincial incidence studies conducted in Canada in 2008 (i.e., Alberta, British Columbia, Saskatchewan and Quebec), there are no known published studies that have specifically examined child welfare practice with respect to infants and infant functioning issues and trends within these unique provincial contexts.

The involvement of infants and their families with the child welfare system is an unmatched opportunity to intervene not only to ensure children’s safety, but also to ameliorate infant development and well-being. The National Scientific Council on the Developing Child [58] suggests that maltreatment should be assessed and treated as a matter of health and development. Thus, when viewed through a developmental lens, these findings raise several questions with respect to the provision of child welfare services to infants and their families: (1) How are current child welfare service models and policies addressing the unique developmental needs of infants? (2) Given that the notion of well-being is a central consideration in child welfare mandates, what alternate or differential response options could best address the needs of infants and their families? And lastly, (3) How can the science of early childhood development inform and enhance child welfare service models? The answers to these questions will guide, and perhaps, re-conceptualize child welfare practice and policy responses with infants and their families.

### Limitations

There are several limitations to this study that must be considered when interpreting the results. The data is representative of an investigative period of 30 days after the case has opened and does not track longer-term service beyond the initial investigation [23]. There may not be enough time for a more comprehensive assessment of child and caregiver functioning, and thus, this may result in the under identification of functioning concerns for either children and/or their caregivers. The exclusion of many predictor variables in the final model may be a result of small sample sizes in many categories; thereby, reducing the possibility of their inclusion in the CHAID analyses and their impact on the outcome variable. The OIS only tracks reports that are investigated by the child welfare system; therefore, reports that are screened out, cases that were not reported, or cases investigated by police alone are not included in this study [23].

## Conclusion

This study presents important findings that contribute to the minimal literature on child welfare service provision to infants and their families. The findings of this research are yet another reminder of the importance of effective targeted prevention and early intervention strategies for this particularly vulnerable subgroup of children and their families. It is critical that child welfare intervention efforts be predicated upon understanding infants’ profiles, developmental vulnerabilities, protective factors, and the risks faced in multiple ecologies. This study underscores the need for future research that contributes to expanding our understanding of the child welfare systems’ responses to infants. Moreover, future research can help to inform, develop and target services to the unique needs of infants and families involved with the child welfare system.

## Abbreviations

IPV: intimate partner violence
OIS: Ontario Incidence Study of Reported Child Abuse and Neglect
